# A counterview of ‘An investigation of the false discovery rate and the misinterpretation of *p*-values’ by Colquhoun (2014)

**DOI:** 10.1098/rsos.150217

**Published:** 2015-08-19

**Authors:** D. Loiselle, R. Ramchandra

**Affiliations:** 1 Department of Physiology, The University of Auckland, Auckland, New Zealand; 2 Auckland Bioengineering Institute, The University of Auckland, Auckland, New Zealand

In commenting on the instructive, comprehensive and entertainingly written article by Prof. Colquhoun (hereinafter referred to as ‘the author’), we state unequivocally that we have no truck with its motivation. Indeed, we too often find the Fisherian approach troubling. Nor do we wish to become involved in the relative merits of ‘Bayesian’ versus ‘Fisherian’ methods. Rather we wish to focus on the author's underlying model, reminding the reader that the output of any mathematical model is only as good as its input parameters. In this regard, we find the numeric value of 0.1 for the parameter describing ‘the probability that the putative effect is real’ to be wholly unrealistic for divining the appropriate *p*-value to be used as the basis for deciding whether the outcome of an experiment provides evidence ‘for’ or ‘against’ rejection of the null hypothesis.

We readily admit that we have no more idea than does the author regarding the true value of the ‘prevalence’ parameter for experimental science, so we have adopted three distinct approaches to estimate it. (i) First, and with reference to the author's charmingly apposite introductory quote from George Elliot's *Middlemarch*, we state our ‘gut instinct’ estimate to be ‘greater than 50%’. (ii) Second, and widening the scope, we have canvassed the senior investigators in our Department of Physiology for their personal estimates of the fraction of times that their explicit, experimentally testable hypotheses have proven to be supported by experimental results. We are aware of the somewhat circular logic of this undertaking because, in each case, ‘classical’ hypothesis testing underlies the ‘guesstimates’. Nevertheless, we consider that well-informed scientists can do better than a flip-of-the-coin (and certainly better than the roll of a decahedral die) in guessing the pathway along which truth lies. (iii) Finally, we have examined the statistical analyses of a selection of published papers (*N*=25), predominantly in the field of ‘cardiovascular biology’ (our personal areas of interest). Our criteria for selection of papers were as follows: (i) those listed in PubMed and published or pre-published during the months of March or April 2015, and available in full, sans cost; and (ii) those in which an experimentally testable hypothesis was either explicitly stated or strongly implied (‘we hypothesize’, ‘we infer’, ‘we propose’, ‘we aim to test’, etc.). We rejected reviews, meta-analyses, case studies, investigations of genetic associations and those articles of a purely descriptive nature. That is, we focused exclusively on studies based on experimental interventions. In all 25 cases, the Fisherian approach had been adopted by the authors, with the value of *α* either stated explicitly or implied in *Results* to be 0.05. Analyses of variance and *t*-tests prevailed. In 14 cases, the authors reported *p*-values of <0.05:<0.02,<0.01,<0.001 or <0.0001 (in one novel case, as an undefined sequence of asterisks embedded in graphs presented in *Results*). References 1 to 25 were the articles we surveyed.

The outcomes of both surveys were surprising but comparable. The ‘guesstimates’ of predictive success by our senior investigator co-workers (*N*=11) ranged from 50% to 90% with the mean ± standard deviation of 69.6% ± 13.1%. With respect to the ‘literature survey’, in only three cases were the authors obliged to state that their results did not support their explicitly stated hypothesis—i.e. the null hypothesis could not be rejected or, in plain English, the authors' scientific hypothesis was declared to be wrong. The complement (22 manuscripts in each of which the null hypothesis was rejected) represents a ‘prevalence’ of 0.88 (a value that exceeds even *our* (probably inflated) ‘gut feelings’).

How are these apparently convincing results to be explained *vis-à-vis* Prof. Colquhoun's counter-conclusion? Do they represent yet another example of publication bias 26; 27 (across some 15 different Journals and journal Editors)? Or have all the investigators succumbed to one or more of: HARKing 28, file drawer-ing 29; 30, under-powering 31; 32, data-stretching 33, bias 30, over-interpretation, *p*-stretching, Bayes-watching 34 or any of the other sins of which hypothesis-testing is accused? It seems unlikely that ‘circular reasoning’ (reflecting the unavoidable fact that, in every case, classical hypothesis testing provided the decision-basis) could have played a large role, especially given that 14 of the results would have satisfied Berger's maximum-likelihood criterion (see appendix A5 of Colquhoun 32). Perhaps we are all unwitting players in a great academic hoax. In this regard, we find it noteworthy that granting agencies commonly favour the presentation of results from ‘pilot studies’. These require the submitter to walk a narrow path between necessarily few observations while avoiding any hint that the study has already been performed. Do such ‘pre-nuptials’ simultaneously dupe both the benefactor and the academic mendicant?

Instead of such speculation, we find it instructive to present an analysis (appendix 1) and graph (figure 1), based on Prof. Colquhoun's ‘tree diagrams’ (figures 2 and 3 in the original).

**Figure 1.**
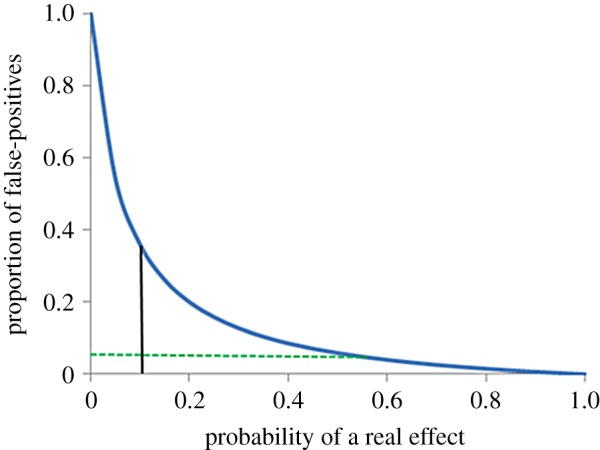
Proportion of ‘false-positive’ rejections of the null hypothesis as a function of the probability that the hypothesized effect exists (i.e. is ‘real’). The curve is drawn for the case when *α* (the ‘significance level’ or the putative risk of a type I error) is 0.05 and the power of the test (i.e. the probability of correctly rejecting the null hypothesis when it is false) has the value 0.8 (mimicking the value adopted by Colquhoun 32).

In figure 1, the vertical line at 0.1 intersects the curve at a value of 0.36, thereby duplicating the data shown in figure 2 of Colquhoun 32. Its location is predicated on the author's implied assumption that biomedical scientists make correct predictions only some 10% of the time. The dashed horizontal line intersects the curve at a value of 0.55. That is, if a scientist makes hypotheses that are correct at least 55% of the time, then he or she is, in fact, already working at the commonly assumed ‘significance’ level of 0.05 (given by the intercept on the ordinate), so that there would be little justification for its 50-fold reduction, as advocated by Prof. Colquhoun. This is perhaps not unexpected, given that nearly 50% of the 25 papers that we surveyed report *p*-values very much smaller than their pre-assigned values of *α*. Furthermore, it accords with the other two of our admittedly ‘free-form’ estimates. Finally, we note that Prof. Colquhoun has examined a specific case of a *p*-value close to 0.05. Our investigation of this issue (performing simulations using Prof. Colquhoun's R-based software program) leads us to conclude that the resulting false discovery rate is likewise dependent on input parameters (especially the critical effect size). Because we wish to maintain focus strictly on the input parameter: ‘prevalence’, we present the results of that investigation in appendix 2 in the electronic supplementary material.

In conclusion, we find it difficult to imagine how science could have achieved its manifold successes if scientists have been wrong 90% of the time. Hence, we suspect that a number of behaviours facilitate a high probability of a real effect, thereby rendering scientific hypotheses robust against extreme probabilities of failure. We count among these behaviours the following common practices: achievement of familiarity with the literature and relentless self-criticism, together with willingness to test ideas in the crucible of public debate, to seek direction from the outcome of under-powered pilot studies, to exploit the power of even simple mathematical models and, on occasion, to disregard much of the preceding and, instead, ‘to go with one's gut-feeling’.

## Supplementary Material

“What happens if we consider p = 0.05, rather than p ≤ 0.05?” This file extends the simulations published in Professor Colquhoun's original manuscript, thereby allowing us to question his conclusion.

## Appendix 1. Derivation of the false discovery rate

Let

*x*= the unknown (and unknowable) probability of a real effect (*P*_real_): 0≤×≤1,
*y*(*x*)= the proportion of false-positive decisions (*F*^+^),
*F*^−^= the proportion of false-negative decisions =*β*×*P*_real_,
*T*^+^= the proportion of true-positive decisions =(1−*β*)×*P*_real_
*T*^−^= the proportion of true-negative decisions =(1−*α*)×*P*_real_; and
*F*^+^= the proportion of false-positive decisions =*α*×(1−*P*_real_),

where *α*= the probability of a type I error (the probability of falsely declaring a true null hypothesis false) and *β*=the probability of a type II error (the probability of failure to reject a false null hypothesis).

In strict accord with the procedure outlined in figure 2 of Colquhoun 32, where it is labelled ‘the false discovery rate’, the proportion of false-positive decisions is given by

$$y(x)=\frac{{\text{F}}^{+}}{\left({\text{F}}^{+}+{\text{T}}^{+}\right)}.$$
