# Supplementary material for: A counterview of ‘An investigation of the false discovery rate and the misinterpretation of p-values’ by Colquhoun (2014)
Source: R Soc Open Sci. 2015 Aug 19;2(8):150217. doi: 10.1098/rsos.150217 (PMC4555854; doi:10.1098/rsos.150217)
Supplement: “What happens if we consider p = 0.05, rather than p ≤ 0.05?” This file extends the simulations published in Professor Colquhoun's original manuscript, thereby allowing us to question his conclusion. [file rsos150217supp1.docx]

**Supplementary Material “What happens if we consider p = 0.05, rather than p ≤ 0.05?”**

Professor Coquhoun posed the question, repeated in the title above, in Section 10 of his paper and addressed it by running repeated simulated t-tests, restricting attention only to those that yielded a p-value in the range 0.045 to 0.05 in order to focus on a specific value: p = 0.047. He seemed to express some hesitancy regarding this approach: “Arguably, this is what we need to do in order to interpret a single experiment that produces p = 0.047” (page 9).

Two simulations were run, each comprising 100,000 tests. The value of *n* for both simulations was 16, thereby achieving approximately 80% power. The critical effect size was unity for the first simulation and zero for the second. The outcomes were 1424 true positives and 511 false positives, respectively (leading to a false discovery rate of 26%), and the conclusion: “Thus, if you observe p ~ 0.05 and declare that you have discovered a real effect, you will make a fool of yourself 26% of the time, even in the most optimistic case.”

We admit to being uneasy about what seems to be a rather arbitrary revision of ‘criterion for statistical significance’ and we are left pondering whether ‘closeness to α’ is one-sided or symmetrical. More seriously, what is the appropriate range of p-values that determines ‘closeness to alpha’? In order to examine this question, we quantified the behaviour of p-values over the complete range from 0.00 to 0.05, making use of Professor Colquhoun’s R-programme, duplicating his procedure, and using his parameters (n = 16 so that power ~ 78%). Two sets of 100,000 iterations were performed, one with δ = (μ_1_ – μ_2_)/σ = 0 (simulating a true null hypothesis) and one with δ = 1 (simulating a false null hypothesis). We divided the range 0.00 to 0.05 into 10 equally-spaced bins, each ranging from P_low_ to P_high_. There are two situations to consider.

Null Hypothesis TRUE (δ = 0)

The total number of true positive results was bin-independent, averaging 78,162, whereas the number falling between P_low_ and P_high_ was highly bin-dependent. The bin-by-bin ratio of the latter number to the former number represents the incidence of true positive tests within a bin, expressed as a fraction of total number of true positive tests. It is shown in Fig S1 by the green triangles.

Null Hypothesis FALSE (δ = 1)

The total number of false positives was approximately bin-independent, averaging 4969, as expected for α = 0.05. Since the number falling in each bin was likewise approximately bin-independent, averaging 491, their ratio was also effectively bin-independent as shown by the red squares in Fig S1. We admit to finding this particular bin-independence counter-intuitive.

The bin-by-bin False Discovery Rate (FDR) is shown in Fig S1 by the blue diamonds. Note that its value in the bin spanning 0.045 to 0.05 is 0.259, consistent with the value of 26% reported by Professor Colquhoun for this range that includes his selected value of 0.047.

Figure S1. False discovery rate (blue symbols) and incidences of false positives (red symbols) and true positives (green symbols) in ten equally-spaced probability bins between 0.00 and α = 0.05.

By forming the bin-by-bin product of the values represented by the blue diamonds, with the incidence of a positive test within a bin (irrespective if they are true or false we arrive at a bin-independent estimate of the incidence of false discovery. Its average value per bin is 0.006. Hence, by summing this value across the ten bins, we arrive at a value of 0.06, in accord with the message conveyed by Fig 1 of the text.

Finally, we note that the incidence of true positive tests is also parameter-dependent, its value within a bin depending on the critical size effect. If the critical size effect is large, then one would expect a greater number of positive p-values in the bin range from 0.005 to 0.01. Contrariwise, if the critical size effect is small, then more positive tests in the range from 0.045 to 0.05 would be expected. Given that no one has any idea of the true effect size prior to undertaking an experiment, we conclude that it is difficult to predict the FDR with accuracy. Hence, if the input parameter δ > 1, when all other parameters remain the same, then the above value of FDR would be reduced. Given this dependence on effect size, it is not correct to state that “26% is the smallest False Discovery Rate possible” even with a prevalence of 0.5.
